# Erratum

**DOI:** 10.1590/1806-9282.20230798ERRATUM

**Published:** 2024-11-11

In the manuscript "Effect of comfort theory-based nursing care on pain and comfort in women undergoing hysterosalpingography: a randomized controlled trial", https://doi.org/10.1590/1806-9282.20230798, published in the Rev Assoc Med Bras. 2023;69(12):e20230798:

On page 3, 1nd column, 4th paragraph:

**Where it reads:**

Kolcaba developed Glasgow Coma Scale (GCS) in 1992 to determine individuals’ comfort requirements and evaluate nursing interventions providing and improving patient comfort19.

**It should read:**

Kolcaba developed General Comfort Scale (GCS) in 1992 to determine individuals’ comfort requirements and evaluate nursing interventions providing and improving patient comfort19.

On page 5, Table 3, 1nd column, 1th line:

**Where it reads:**

Glasgow Coma Scale

**It should read:**

General Comfort Scale

